# The Role of Retinoic-Acid-Related Orphan Receptor (RORs) in Cellular Homeostasis

**DOI:** 10.3390/ijms252111340

**Published:** 2024-10-22

**Authors:** Darya Nematisouldaragh, Eryn Kirshenbaum, Michael Uzonna, Lorrie Kirshenbaum, Inna Rabinovich-Nikitin

**Affiliations:** 1 Department of Physiology and Pathophysiology, Rady College of Medicine, Max Rady Faculty of Health Sciences, University of Manitoba, Winnipeg, MB R2H 2A6, Canada; dnemati@sbrc.ca (D.N.); ekirshenbaum@sbrc.ca (E.K.); muzonna@sbrc.ca (M.U.); lkirshenbaum@sbrc.ca (L.K.); 2 The Institute of Cardiovascular Sciences, St. Boniface Hospital Albrechtsen Research Centre, Winnipeg, MB R2H 2A6, Canada; 3 Department of Pharmacology and Therapeutics, Rady College of Medicine, Max Rady Faculty of Health Sciences, University of Manitoba, MB R2H 2A6, Canada

**Keywords:** ROR, cell death, autophagy, mitochondria, cardiovascular disease, inflammation, ROS

## Abstract

Retinoic-acid-related orphan receptors (RORs) are transcription factors belonging to the nuclear receptor subfamily consisting of RORα, RORβ, and RORγ. By binding to the ROR response elements (ROREs) on target gene promoters, RORs regulate a wide variety of cellular processes, including autophagy, mitophagy, oxidative stress, and inflammation. The regulatory roles of RORs are observed in cardiac cells, hepatocytes, pulmonary epithelial cells, renal cells, immune cells, and cancer cells. A growing body of clinical and experimental evidence suggests that ROR expression levels are markedly reduced under different pathological and stress conditions, suggesting that RORs may play a critical role in the pathogenesis of a variety of disease states, including myocardial infarction, immune disorders, cancer, and metabolic syndrome. Reductions in RORs are also associated with inhibition of autophagy, increased reactive oxygen species (ROS), and increased cell death, underscoring the importance of RORs in the regulation of these processes. Herein, we highlight the relationship between RORs and homeostatic processes that influence cell viability. Understanding how these intricate processes are governed at the cellular level is of high scientific and clinical importance to develop new therapeutic strategies that modulate ROR expression and disease progression.

## 1. Introduction

The nuclear receptor (NR) superfamily belongs to a family of related transcription factors that regulate a variety of physiological and pathophysiological processes, including embryonic development, metabolism, and inflammation [1,2]. NRs are classified into three distinct groups: non-steroid hormone receptors, steroid hormone receptors, and nuclear orphan receptors [3]. Retinoic-acid-related orphan receptors (RORs) belong to the nuclear receptor subfamily that consists of RORα, RORβ, and RORγ [2]. RORs bind as monomers to the cis-acting ROR response elements (ROREs) on target gene promoters. These orphan receptors recognize the consensus core motif AGGTCA flanked by 5′ followed by a 5-bp A/T-rich sequence [4]. Structurally, RORs comprise a highly conserved DNA-binding domain (DBD), a ligand-binding domain (LBD), a hinge domain, and a diverse N-terminal domain [5]. Through the utilization of distinct promoters and/or alternative splicing mechanisms, RORs create multiple isoforms which are characterized only by their N-terminal region [6]. The mapping of RORs indicates that these proteins have distinct areas on the human chromosome, with RORα being mapped to 15q22.2, RORβ to 9q21.13, and, lastly, RORγ to 1q21.3 [7].

**Figure 1:**
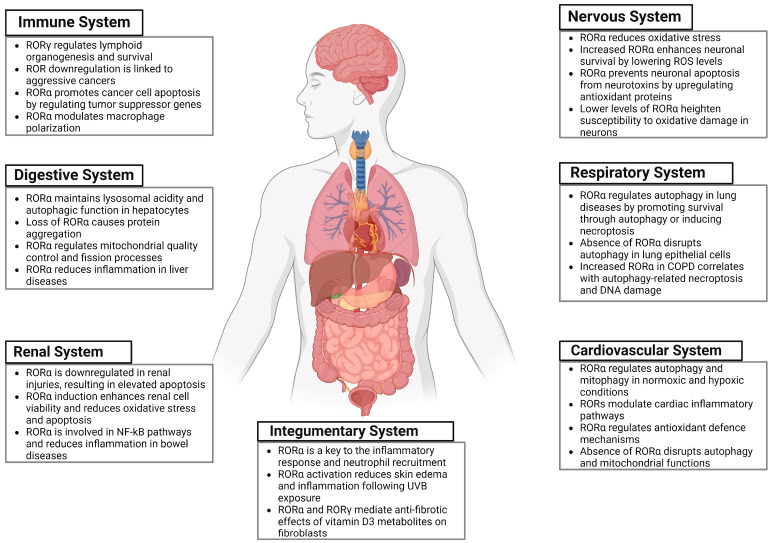
Schematic representation of organ systems where RORs play a role in the death mechanisms of cellular homeostatic processes.

Many physiological processes and biological functions are regulated by RORs (Figure 1). These include cellular differentiation, brain development, circadian rhythms, immune system, cardiovascular system, and lipid metabolism [7,8,9]. The regulatory roles of these proteins depend on their ability to act as repressors or activators of gene transcription through the interaction with corepressors and coactivators [7]. Many studies over the past several years have shown that RORs can exert a protective function in different organ systems. For example, RORα has been shown to exert cardioprotective effects following hypoxic stress through adaptive quality control processes, such as autophagy [8]. Additionally, RORα has also been shown to protect against cardiac hypertrophy and heart failure induced by the renin–angiotensin–aldosterone system (ANG II) [10]. In patients suffering from diabetic cardiomyopathy, RORα has been found to reduce oxidative stress, apoptosis, and autophagic dysfunction, thereby leading to the attenuation of disease progression and cardiac dysfunction [11].

Further, RORs are also involved in brain function and neuroprotection through a mechanism that reduces oxidative stress. This has been shown in animal studies, demonstrating that overexpression of RORα in neurons with disrupted redox homeostasis may lead to decreased reactive oxygen species (ROS) production, thus increasing the neuronal survival rate [12]. Interestingly, RORα has also been implicated in behavioral disorders such as autism. This has been demonstrated by finding reduced RORα expression levels in individuals with autism spectrum disorder (ASD), coincident with a decline in ROR target gene expression [13,14]. Activation of RORα has further been shown to cause a reduction in repetitive behavior in mice models and an increase in RORα target gene expression in both human cell lines and mice models [13].

Notably, RORs are further engaged in the pathophysiological functions of other diseases, including immune disorders, cancer, and metabolic syndromes. Findings have demonstrated that RORγ is required for lymphoid organogenesis and thymocyte survival. Loss of RORγ leads to declines in the antiapoptotic factor Bcl-xL and in the precursor cells of lymphoid organs [15,16]. Additionally, RORγ knockout results in the development of lymphoblastic lymphoma in adult mice [15]. These studies suggest that RORγ is an essential regulator of the immune system and that it is crucial for inhibiting lymphoblastic lymphoma. Further research has also demonstrated the role of RORγ in the regulation of tumorigenesis. RORγ expression is downregulated in patients suffering from aggressive basal-like breast cancer and bladder cancer, supporting the important modulatory role of RORγ in the regulation of carcinogenesis and tumor suppression [3,17]. Activation of RORs by nobiletin, a polymethoxylated flavonoid and ROR activator, has been found to enhance the antitumor properties of RORs in a triple-negative breast cancer (TNBC) model by suppressing the NF-κB signaling pathway [18].

**Figure 2:**
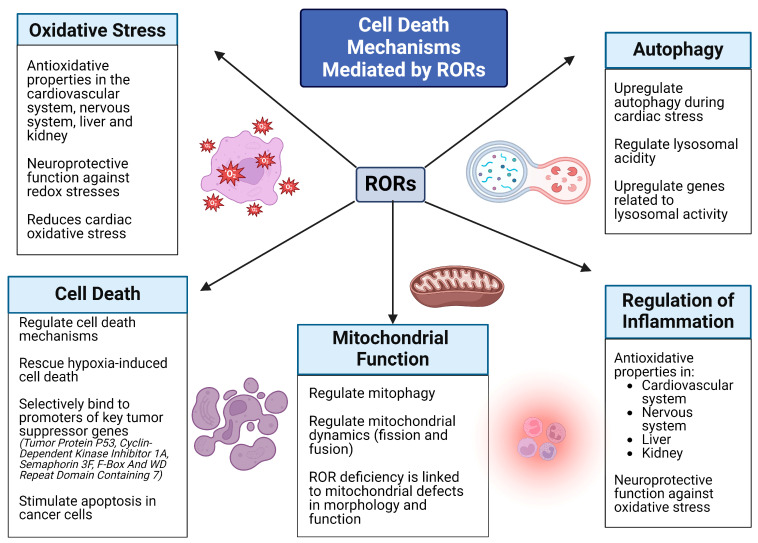
Summary of the different roles of RORs in cell death mechanisms.

RORs exhibit key protective roles in the development and progression of various human diseases through the regulation of oxidative stress, cellular quality control processes, and cell death mechanisms. Therefore, in this review, we highlight the recent advances and developments in the research of ROR-dependent cell death and cell survival mechanisms that underlie pathologies of various organ and tissue systems (Figure 2).

## 2. The Roles of RORs in Autophagy

Autophagy is a highly conserved process whereby damaged proteins and organelles are degraded through an elaborate autophagosome–lysosome signaling pathway that is crucial for maintaining cellular homeostasis. Adaptive autophagy is induced during cellular stress, such as nutrient stress, inflammation, ER stress, and hypoxia [19,20]. When autophagy is disrupted, it leads to accumulation of misfolded proteins and damaged organelles such as mitochondria. This, subsequently, leads to the activation of cell death mechanisms [21,22]. For this reason, alterations to the autophagy pathway are linked to the development of various human pathologies, including cancer, cardiovascular diseases, neurodegenerative diseases, and metabolic disorders [19,22]. In some contexts, however, these changes may also have a protective role, reflecting the intricate balance that autophagy maintains in healthy and diseased subjects.

RORs have been shown to play a crucial role in the regulation of autophagy under both basal and stress conditions. In the heart, impaired autophagy leads to the death of cardiac myocytes, resulting in cardiac remodeling and dysfunction in a variety of cardiovascular diseases, including MI, doxorubicin-induced cardiomyopathy, and heart failure [23,24,25]. Recent studies have identified a close link between autophagy regulation during hypoxic stress and RORα. Reduced levels of RORα during hypoxia have been found to result in increased cardiac cell death from disrupted autophagy and increased mitochondrial perturbations [8]. Interestingly, upregulation of RORα induced by nobiletin has been shown to restore autophagic function, reduce ROS-producing mitochondria, and increase the survival of cardiac cells following hypoxia [8]. Furthermore, in in vivo mouse models, reduced levels of RORα, followed by inhibition of the autophagy flux, have also been observed in subjects suffering from diabetic cardiomyopathy and in association with diastolic dysfunction and cardiac remodeling of diabetic hearts. In this study, a reduction in autophagosomes coincided with the activation of apoptosis and increased oxidative stress by reducing the expression of antioxidant genes, a phenomenon which was reversed through pharmacological activation of RORα by melatonin and SR1078 [11].

RORα plays a crucial role in the upregulation of autophagy and mediation of lysosomal acidity in hepatocytes [26]. Lysosomes are vesicles that contain acid-rich hydrolases and play a pivotal role in autophagy and phagocytosis. In fact, these membrane-bound organelles serve as the final degradation site for autophagy, where the autophagosome cargo is degraded [27]. Lysosomes sustain cellular homeostasis by establishing a highly acidic environment with pH levels ranging between 4.5 and 5.0. The hydrolytic enzymes within the lysosomes are responsible for the breakdown of engulfed biological molecules and for the recycling of extracellular and intracellular substances [27]. The disruption of lysosomal function, specifically through the impairment of its acidification, can lead to disease onset and progression [27]. In this regard, a recent study has found that the absence of RORα in hepatocytes decrease lysosomal acidity [26]. In contrast, administration of an adenoviral vector encoding RORα was found to rescue the lysosomal pH in the same study [26]. Furthermore, in the absence of RORα, aggregation of LC3-II, p62, and neighbor of BRCA1 gene 1 (NBR1) was detected. However, induction of RORα was found to increase the expression of genes related to lysosomal activity, including the *Atp6v1g1* gene which codes for a subunit of vacuolar H^+^-ATPase [26]. This study highlights the importance and role of RORα in the maintenance of lysosomal acidification properties and autophagic function in the liver.

A link between RORα and autophagy has also been established in relation to human lung diseases. While autophagy primarily functions as a protective mechanism, preserving cellular homeostasis and, potentially, preventing cell death, it can also modulate other maladaptive pathways of cell death, including autophagy-dependent necroptosis, which may contribute to the pathogenesis of chronic obstructive pulmonary disease (COPD) [28]. Cigarette smoking, ageing, and environmental factors have all been associated with autophagic dysfunction, ROS production, aggregation of misfolded proteins, cell senescence, and cell death [29]. In COPD, cigarette smoke triggers epithelial cell death and extrinsic apoptosis activated by maladaptive autophagy, resulting in the destruction of the alveoli and the development of emphysemas [30,31]. Interestingly, patients with COPD exhibit higher RORα expression, which is associated with increased DNA damage in their lung tissues [32]. Furthermore, RORα and autophagy levels have been shown to be upregulated in human lung epithelial cells and in primary fibroblasts subjected to cigarette smoke extract. However, the absence of RORα has been shown to inhibit autophagy and autophagosome formation in lung epithelial cells [33]. These findings signify a dual role for RORα in patients suffering from lung diseases, suggesting that, unlike in MI, upregulation of RORα in lung cells can modulate autophagy to induce cell death. Therefore, future research endeavors exploring cigarette-smoke-induced RORα activation and its role in modulating autophagy in cardiac tissue may provide valuable insights for potential therapeutics.

Studies have also demonstrated the mechanistic roles of RORα in diabetic cardiac tissues and epithelial cells of the proximal tubules during renal I/R injury diabetes by demonstrating that RORα levels are reduced in these pathologies [11,34]. In relation to diabetes, the absence of RORα has been found to markedly heighten diastolic dysfunction and cardiac remodeling in in vivo animal models [11]. This has been shown to be linked to exacerbated myocardial apoptosis, dysfunctional autophagy, and elevated oxidative stress caused by disruption of antioxidant genes [11]. Oxidative stress is the imbalance between ROS production and the ability of antioxidant mechanisms to neutralize or repair the resulting damage. RORα has been shown to be downregulated in mouse hearts following myocardial ischemia/reperfusion (I/R), but not RORγ [35]. In this particular study, the imperative role of RORα in the protection against I/R injury was verified in RORα-deficient mice subjected to MI/R injury [35]. Deficiency in RORα was found to lead to a significant increase in the magnitude of myocardial infarcts, marked elevation of myocardial apoptosis, and aggravated contractile dysfunction [35]. In addition, RORα deficiency also triggered MI/R-induced endoplasmic reticulum stress, impaired mitochondrial function, and autophagic activity [35]. Similarly, renal I/R injury has also been found to be exacerbated in kidney cells in the absence of RORα in both animal studies and human proximal tubule cell lines [34]. In this last study, RORα deficiency resulted in renal dysfunction and increased kidney morphological damage [34]. Altogether, these studies substantiate the inherent protective role of RORα in the mitigation of cardiovascular diseases and other injuries and highlight the importance of RORα with respect to the regulation of autophagy and preservation of cellular homeostasis.

## 3. The Roles of RORs in Oxidative Stress

ROS are produced in different organelles, including the mitochondria, endoplasmic reticulum, plasma membrane, and nucleus. Excessive ROS production is associated with cerebral stroke, MI injury, and neural traumas. RORα has been suggested to display antioxidative properties. Specifically, RORα1, an isoform of RORα, is believed to be neuroprotective against redox stresses. Cortical neurons exposed to β-amyloid peptide (Aβ), C2-ceramide, and H_2_O_2_ increase ROS and apoptosis [36]. However, overexpression of RORα1 in such neurons upregulates antioxidant proteins, peroxiredoxin 6, and glutathione peroxidase 1 [36]. In the context of cardiovascular diseases, RORα is cardioprotective owing to it having antioxidant properties. In diabetic hearts, RORα regulates the antioxidant defense mechanism and reduces cardiac oxidative stress [11]. Similarly, RORα reduces ROS production in heart tissues after MI/R and angiotensin II (ANGII) stress [10,35], as well as in cardiac myocytes following hypoxic stress [8]. The absence of RORα is correlated with elevated oxidative stress in diabetic hearts, evidenced by a significant increase in ROS accumulation, nitrotyrosine production, and apoptosis [11]. Furthermore, the absence of RORα significantly downregulates the expression of antioxidant genes, a phenomenon which can be reversed through upregulation of RORα which, in turn, mitigates cardiac dysfunction and remodeling through the suppression of apoptosis, rescue of autophagy, and reduction of oxidative damage [11]. Moreover, RORα deficiency also significantly augments oxidative stress in rodent heart tissues after MI, evidenced by the elevated levels of ROS and nitrotyrosine content [35]. Notably, the damage caused by oxidative stress due to RORα deficiency has also been observed in heart tissues with ANG-II-induced diseases [10]. However, induction of RORα in the ANG II hearts was found to prevent cardiac hypertrophy [10].

The function of RORα as an antioxidant is also evident in the liver. Elevated levels of hepatic oxidative stress and inflammation are the predominant drivers of nonalcoholic steatohepatitis (NASH). Furthermore, RORα levels are markedly reduced in individuals with liver steatosis [37]. In addition, one of the main mechanisms underlying fatty liver disease includes increased mitochondrial ROS production, elevated apoptosis, and lipid peroxidation [38]. Interestingly, the antioxidative role of RORα, decreasing ROS levels and diminishing lipid peroxidation in the liver, has been shown in primary hepatocytes and Kupffer cells, where RORα upregulation increases mRNA levels of enzymes involved in antioxidant defense mechanisms, superoxide dismutase 2 (SOD2), and glutathione peroxidase 1 (GPx1) [39]. These enzymes are involved in the neutralization of superoxide radicals and ROS. Treatment with JC1-40, an activator of RORα, diminishes diethylnitrosamine-induced acute liver injury, further confirming the defense mechanism of RORα against oxidative stress in NASDH models in mice [39].

Hypoxic and oxidative stress are also implicated in various kidney diseases, including acute kidney injury [40,41]. RORα is downregulated in proximal tubules, in the cortex, and in the outer medulla of the kidney during renal I/R injury and post I/R, consistent with reduced RORα levels. In the absence of RORα, oxidative stress levels are elevated, together with increased epithelial cell apoptosis and renal tubular injury after I/R. Interestingly, upregulation of RORα by pharmacological agonists augments renal viability, inhibits epithelial cell apoptosis, and suppresses oxidative stress induced by renal I/R in the ischemic kidney [34]. These studies provide a novel understanding of the inherent roles of RORs as antioxidants in different tissues and organs and present new perspective on the defense mechanisms of RORα in oxidative stress.

## 4. The Role of RORs in Mitochondrial Biogenesis/Function

The coordinated interplay among mitochondrial dynamic mechanisms orchestrates the preservation of mitochondrial homeostasis. These processes include the synthesis of new mitochondria through mitochondrial biogenesis and the clearance of damaged mitochondria via mitophagy, a specific form of mitochondrial autophagy [42]. Disrupted mitochondrial dynamics and function are linked to cardiovascular diseases, neurodegenerative diseases, and type-2 diabetes [42]. Notably, recent studies have demonstrated that RORα is integral to mitochondrial function in cardiac myocytes [43]. RORα modulates mitophagy in cardiac myocytes under both normoxic and hypoxic conditions, a phenomenon which is pivotal for the maintenance of mitochondrial bioenergetics, morphology, and abundance [43]. In addition, following ANG II treatment, RORα preserves the function of mitochondria and alleviates oxidative stress [10]. Interestingly, stimulation of RORα by nobiletin upregulates mitophagy and reverses hypoxia-induced mitochondrial perturbations and impairment [8]. Furthermore, induction of RORα restores mitochondrial oxygen consumption rate (OCR) during hypoxia, resulting in increased viability of cardiac cells [8].

Inhibition of RORα is linked to defects in mitochondrial morphology and function. In diabetic mice, RORα deficiency has been shown to not only increase apoptosis, but also lead to elevated mitochondrial aggregation and disorganization of the cristae structure [11]. Additionally, in RORα knockout mice fed a high-fat diet (HFD), a decrease in mitochondrial density and biogenesis, together with structural changes and mitochondrial dysfunction, have been observed [44]. In the heart, RORα knockout results in reduced mitochondrial abundance and impaired mitochondrial structure and function [43]. This can be associated with reduced contractility, elevated oxidative stress, increased cell death, and decreased autophagic flux [43].

Notably, RORα is involved in the caveolin-3 (Cav-3) pathway, which is one of the several pathways that regulate mitophagy [43]. RORα has been shown to induce transcription of Cav-3 in cardiac cells [43], as well as in skeletal muscle cells [45]. In silico analysis has revealed two consensus RORE sequences on the *Cav3* gene promoter, suggesting that RORα can bind directly to the Cav-3 promoter to modulate mitophagy under both normoxic and hypoxic conditions.

The regulatory effect of RORα on mitochondrial dynamics is also evident in the liver. RORα knockout mice fed an HFD demonstrate reduced expression of mitochondrial oxidative phosphorylation (OXPHOS) proteins, including NADH dehydrogenase 1 beta subcomplex subunit 8 (NDUFB8) and ATP synthase in the liver [46]. RORα deficiency also impairs mitochondrial fission in hepatocytes, leading to abnormalities in mitochondrial quality control [46]. In the liver of steatohepatitis patients, the levels of Bnip-3 and PGC-1α are reduced, a phenomenon that is consistent with lower levels of RORα [46]. However, overexpression of RORα reverses mitochondrial fission through upregulation of fission proteins [46]. These studies suggest that RORs play key roles in mitochondrial quality control and bioenergetics processes in various organs, a phenomenon which may in part explain the protective role of RORα through inhibition of cell death.

## 5. The Role of RORs in Inflammation

Acute inflammation during injury or disease activates a series of molecular mechanisms that lead to signaling exchange and activation of the cellular defense network in order to mitigate the outcomes of injury or infection [47]. However, excess or unregulated inflammation can lead to a chronic disease [47]. Studies have revealed a link between regulation of different isoforms of RORs and immune responses [48]. RORC has two isoforms, RORγ1 and RORγt, of which RORγt is highly expressed in various types of immune cells, including thymocytes, T helper cells, and lymphoid cells [49]. RORs are also required for T cell lineage specification and T helper cell differentiation [50], organogenesis of secondary lymphoid tissues (lymph nodes and Peyer’s patches) [51,52], regulation of thymopoiesis [7,16,53], lymphocyte development and cytokine production [54], immune homeostasis, and adaptive immune responses.

Notably, RORs play an important role in the intricate regulation of inflammation within the cardiac tissue through the nuclear factor kappa B (NF-κB), the mitogen-activated protein kinase (MAPK), and the JAK-STAT pathway [47]. RORα can repress inflammation by directly inhibiting the transcription of core proinflammatory mediators, IL-6 cytokine, and NF-κB in response to ANG II stress [10]. In addition, RORα also regulates STAT3 Ser^727^ phosphorylation and preserves mitochondrial function against ANG II [10]. Loss of RORα results in greater expression of IL-6 and induces activation of STAT3 and of the NF-κB pathway in response to ANG II. Deficiency of RORα leads to reduced contractility of the heart and heart failure in both human and mouse heart models of hypertrophy [10]. In addition, in neonatal mice models of MI, macrophages exhibit properties of heart regeneration and angiogenesis [55]. Maresin-1 (MaR1) is a macrophage lipid mediator with anti-inflammatory and proresolution properties [56,57,58,59]. In neonatal rat cardiomyocytes, RORα has been shown to regulate MaR1, contributing to the suppression of the inflammatory response [60]. Cardiac hypertrophy is also mediated by MaR1 via RORα through the phosphoinositide 3-kinase (PI3K)/Akt signaling pathway and through the upregulation of IGF-1 [60]. In liver macrophages, MaR1 binds to RORα, demonstrating the crucial regulatory role of RORα in inflammatory processes in liver diseases such as NASH [56]. Other studies have also illustrated the resolving properties of MaR1 in the skin. MaR1 is able to repress skin edema, decrease inflammation and cytokine production, and inhibit neutrophil recruitment in skin injuries in animal models subjected to UVB [61] through RORα-dependent induction of 12-lipoxygenase (12-LOX), which increases MaR1 levels [56].

RORs are also involved in regulating inflammation in the hepatocytes and liver diseases. One of the underlying mechanisms of NASH is related to chronic inflammation, elevated oxidative stress, and apoptosis, all of which contribute to the development of steatohepatitis [39]. Kupffer cells can respond to oxidative stress by inducing inflammation. High ROS levels activate NF-κB and induce the production of cytokines such as tumor necrosis factor α (TNFα) which are involved in the inflammatory response. Overexpression of RORα diminishes the inflammatory cytokine expression and attenuates NASH by increasing the expression of SOD2 and GPx1 in core immune cells, hepatocytes, and Kupffer cells. Another mechanism through which RORα regulates inflammation in the liver is through the polarization of macrophages [56]. M1 Kupffer cells produce cytokines, such as TNFα and IL-1β, which lead to inflammation and pathogenesis of liver steatosis [62]. In contrast, M2 Kupffer cells promote the death of M1 Kupffer cells [63]. Therefore, the inflammation induced by the M1 macrophages is counteracted by the M2 macrophages, which stimulate the resolution of inflammation and facilitate tissue healing [63]. Studies have recently shown that RORα mediates M1/M2 polarization in Kupffer cells and monocyte-derived macrophages [64]. M1/M2 macrophage polarization is an intricately regulated process, involving a network of signaling pathways and transcriptional regulatory mechanisms [65]. Different signaling molecules, including cytokines, can dynamically influence and alter the polarization of macrophages [65]. In fact, the imbalance between M1 and M2 macrophages is correlated with various diseases, inflammatory responses, and cancer [65]. M1 macrophages are characterized by their proinflammatory responses and tumor suppression, while M2 macrophages are associated with anti-inflammatory effects and promote tumor progression [66]. Elevated tumor-associated macrophages—predominantly M2 macrophages, infiltrating the tumor microenvironment—or reduced M1 polarization can mediate neoplasia, including tumor growth and metastasis [66,67]. RORα facilitates the reduction of M1 KC expression levels and mediates M2 KC polarization. This results in decreased inflammation and alleviated symptoms of NASH in mice [64]. Another study has shown that the anti-inflammatory process and the M2 polarity switch are facilitated through the binding of MaR1 to RORα in macrophages and that MaR1 suppresses NASH in HFD-induced mouse models in a RORα-dependent manner [56].

Moreover, RORs play a pivotal role in the progression of melanomas and various types of cancer, significantly influencing tumor growth and immune responses, as well as shaping potential therapeutic outcomes. Primarily, RORα and RORγ expression is diminished in melanoma relative to benign nevi, with further decreases observed in advanced stages and metastatic lesions [68]. Lower levels of RORα and RORγ are associated with more aggressive tumor phenotypes and poorer prognoses, whereas elevated expression correlates with improved survival outcomes [68]. These results underscore the critical role of RORs in melanoma progression [68]. Notably, RORα is a critical transcription factor in the development of cutaneous squamous cell carcinoma (cSCC), significantly influencing its progression [69]. Research has demonstrated that RORα downregulates key proteins involved in cSCC cell growth and migration, such as S100 calcium-binding protein A9 (S100a9) and small proline rich protein 2F (Sprr2f) [69]. Although RORα is inhibited in cSCC, its activation has been demonstrated to inhibit tumor growth, highlighting its potential as a promising target for future cSCC treatments [69]. Dysregulation of RORα and RORγ, both pivotal regulators of antioxidant defense mechanisms in multiple organ systems, including the cardiovascular, nervous, immune, hepatic, and renal systems, has been associated with heightened oxidative stress [8,10,11,34,35,36,37,39,46]. This disruption fosters a pro-oxidant environment by downregulating key antioxidant enzymes, such as SOD2 and GPx1, leading to increased ROS production [39]. The resultant oxidative imbalance promotes genomic instability and accelerates mutation accumulation, thereby facilitating tumor progression and contributing to more aggressive cancer phenotypes.

Additionally, other studies have illustrated that the activation of RORα suppresses aerobic glycolysis and biosynthesis in hepatoma cells [70]. This suppression, linked to RORα-mediated decrease in pyruvate dehydrogenase kinase 2 expression, inhibits hepatoma growth through mechanisms involving the upregulation of p21, as observed in both in vitro and in vivo models [70]. In addition, research highlights that the efficacy of melatonin in inhibiting melanoma growth varies based on the presence of specific receptors, including RORα, and cytosolic proteins present in different human melanoma cell-lines [71]. Interestingly, melatonin’s ability to alleviate oxidative stress offers a multi-faceted approach for the management of malignancies [72,73,74,75]. As a ROR ligand, melatonin not only mitigates oxidative damage by neutralizing ROS and by enhancing antioxidant defenses, but also promotes ROR-mediated mechanisms that induce apoptosis in cancer cells [72,73,74,75,76,77,78]. This integrated effect highlights the potential of ROR ligands with antioxidant properties to support cancer treatments by regulating oxidative balance, driving cancer death pathways, and limiting mutation accumulation. Moreover, specific ROR ligands, such as SR1078, have been demonstrated to inhibit aerobic glycolysis in hepatoma cells, underscoring the therapeutic potential of targeting RORs to intervene in metabolic pathways critical for the survival of cancer cells [70].

Notably, RORγ is also involved in tumor immunity through the regulation of IL-9 production [79]. The absence of RORγ, a critical component of the proinflammatory type 17 T helper (Th17) cell pathway, results in elevated IL-9 production by T cells, a phenomenon which is associated with significant suppression of melanoma growth in murine melanoma models [79]. Blockade of IL-9 diminishes the tumor growth-inhibiting effects, indicating that IL-9, modulated by RORγ, plays a critical role in tumor suppression [79]. Thus, RORs, particularly RORα and RORγ, are critical for cancer progression, especially with respect to melanoma, highlighting their potential as therapeutic targets aimed at tumor suppression.

Furthermore, the anti-inflammatory role of RORα is also evident in the hormone biotransformation pathway, the gastrointestinal system, and the gut microbiome. Human cytosolic sulfotransferases (SULTs) are a family of enzymes that modulate small signaling molecules [80]. They are responsible for transferring a sulfate group from 3′-phosphoadenosine 5′-phosphosulfate (PAPS) to acceptor molecules. These enzymes regulate metabolic processes, and their functions are intricately associated with various pathologies [80]. Studies have discovered that RORs regulate the human hydroxysteroid sulfotransferase (*SULT2A1*), which is a member of the SULT family [37]. RORα and RORγ induce the *hSULT2A1* gene promoter in human liver tissues and hepatocytes; however, loss-of-function studies illustrate that downregulation of ROR genes represses the expression of endogenous *SULT2A1* [37]. RORs exert a positive regulatory influence on the expression of *SULT2A1* in vivo, contrasting with their negative regulation of mouse *SULT2A1*. In the gut environment, a finely tuned equilibrium maintains the interplay between anti- and proinflammatory mechanisms of RORs, thereby sustaining the epithelial barrier and regulating intestinal homeostasis and inflammation. Studies have shown a bidirectional crosstalk between the gut microbiota and various immune cells [81,82,83]. In this regard, RORγt is not only associated with lymphoid tissue organogenesis, but also with the development of immune cells, including Th17 cells and innate lymphoid cells type 3 (ILC3s) [84]. Immune cells expressing RORγt (RORγt^+^) are involved in barrier immunity and tolerance mechanisms against symbiotic microbiota [84]. The underlying mechanism involves spatial segregation of microbes, removal of microbiota-reactive T cells, as well as stimulation of regulatory T cells [84]. However, further research is required to gain a more profound understanding of the involvement of RORs in the immune cell differentiation pathway and its connection with the gut microbiome. Furthermore, RORs also have a protective function in diseases associated with the gastrointestinal tract, such as bowel diseases and ulcerative colitis [5]. In bowel diseases, RORα reduces inflammation by directly interacting with NF-κB and corepressor HDAC3 [85]. The underlying mechanism involves the recruitment of HDAC3 by RORα to inhibit the transcription of NF-κB, thereby terminating the activation of NF-κB target genes. This ROR-mediated NF-κB target suppression leads to reduced inflammation and survival [85]. In addition, through the inhibition of NF-κB, RORα also influences the metabolic changes and the effector reactions in cytotoxic T cells (CD8+ T cells). Furthermore, the antitumor immune response is an essential aspect of the immune system, and cytotoxic cells are critical effectors with respect to antitumor immunity; however, cancer cells have the ability to avoid immune cytotoxicity. RORα repression of NF-κB targets (*Acat1/2* and *Abca1*) induces the production of cytotoxic cytokines, including TNF-α and interferon-γ (IFN-γ) by CD8+T cells [85]. This has prompted further research to explore the signaling pathways involved in the anti-inflammatory function and the physiological role of RORα in the large intestine. Since anti-inflammatory drugs have different immunomodulatory effects [86] and can alter the cytotoxic effector activities with respect to antitumor immune responses, the inherent capability of RORs to suppress inflammation and increase cytotoxic effector function illustrates a great potential for the development of therapeutic strategies for oncological disorders, such as immunogenic tumors, as well as intestinal inflammation.

## 6. The Role of RORs in Cell Death

RORs emerge as regulators of cell fate by navigating the molecular pathways that govern cell death. Knockout of RORα in cardiac cells results in increased cell death not only during hypoxia, but also during normoxia, suggesting the involvement of RORα in the regulation of cardiac cell viability [8]. However, induction of RORα via nobiletin is able to reverse hypoxia-induced cell death under hypoxia [8]. The cardioprotective function of RORα is also evident in patients suffering from diabetic cardiomyopathy, where overexpression of RORα reduces cell death mediated by autophagy dysfunction [11].

Hydrogen sulfide (H_2_S) has been previously suggested as a potential target for treating cardiovascular diseases. In vitro and in vivo studies illustrate that H_2_S can preserve mitochondrial function, reduce oxidative stress, and inhibit necroptosis in a RORα-dependent manner in patients suffering from diabetic cardiomyopathy [87]. Suppression of RORα inhibits the ability of H_2_S to rescue cardiac function and inhibit necroptosis [87]. Interestingly, this study also found that H_2_S can reverse the downregulation of RORα in the diabetic myocardium, and that upregulation of RORα induced by H_2_S facilitates phosphorylation of STAT3 at Serine 727, a phenomenon which suggests that STAT3 could be a downstream target of RORα in the suppression of necroptosis [87]. This suggests that further studies are required to explore the modulation of RORα with respect to STAT3 and the involvement of H_2_S in the modulation of necroptosis in relation to other cardiovascular conditions. Furthermore, since autophagy-dependent necroptosis is one of the major contributors to COPD [28], evaluating this mechanistic avenue may be of interest in the field of pulmonary diseases, and further studies should explore whether the regulation of RORα and the treatment with H_2_S may have favorable outcomes in the epithelial cells of COPD.

Interestingly, RORα has been shown to be beneficial to the stimulation of apoptosis in cancer cells [76,77,78]. One mechanism through which RORs facilitate increased apoptosis in cancer is through stabilization of the tumor suppressor protein p53 [77,78]. In fact, RORα directly regulates p53 by binding to RORE elements on the p53 promoters [78]. P53 is a core protein involved in the government of fundamental cellular functions encompassing DNA repair, cell cycle modulation, cellular integrity, and apoptosis; however, in the majority of human cancers, p53 is mutated, leading to development of cancerous tumors [77]. Absence or abnormal function of p53 triggers uncontrolled cellular growth and division, both of which induce the development of malignancies [77]. In addition to its role in the stabilization of p53, RORα catalyzes the transcriptional activation of p53 through engagement with deubiquitinating enzymes in a HAUSP-dependent mechanism [78]. Subsequently, p53 target genes are activated to directly participate in apoptosis [78]. In addition, other studies have demonstrated that RORα can bind selectively to promoters of other key tumor suppressor genes. These genes include *p21* (p21^Cip1^), *SEMA3F* (semaphorin III/F), and *FBXW7* (F-box and WD repeat domain containing 7) [76]. Reduced levels of RORα and SEMA3F have been found to be linked to poor clinical outcomes in human breast cancer cells; however, RORα activation suppresses aggressive phenotypes, tumor invasion, and growth of breast cancer cells in vivo [88].

*RORC* is also involved in the regulation of cellular division and glucose metabolism [3]. Cao et al. (2019) illustrated that RORC markedly diminish glucose metabolism in cancer cells, elucidating the inhibitory function of RORs with respect to the proliferative properties of cancer cells. Additionally, overexpression of *RORC* sensitizes cancer cells to cisplatin chemotherapy and augments the apoptotic response pathway [3]. *RORC* inhibits STAT3 translocation to the nucleus through the impediment of the programmed death ligand-1 (PD-L1)/integrin β6 (ITG β6) signaling cascade. This further suppresses cancer cell proliferation and elevation of apoptosis triggered by cisplatin in vivo [3]. Furthermore, animal studies and human cell line assays of cSCC reveal that RORα also inhibits cell proliferation and migration of this type of cancer [69]. Activation of RORα mitigates cancer progression by decreasing invasiveness and proliferation in both human and mouse cSCC cell lines [69]. Interestingly, ROR expression levels are downregulated in tumor tissues of bladder cancer, breast cancer, colorectal cancer, gastric cancer, hepatocellular carcinoma, and cutaneous squamous cell carcinoma [3,37,69,76,88,89]. Downregulation of RORs in cancer cells is linked to disease progression and adverse prognostic outcomes [76]. One of the pathways that contribute to the reduction of RORα in cancer cells is reduced activation of AMP-activated protein kinase (AMPK) [76,90]. Treatment with the AMPK activator AICAR results in a substantial elevation of RORα levels in cancer cells [76]. Concurrently, activation of AMPK increases translocation of RORα to the promoters of tumor suppressor genes, increasing induction of apoptosis in cancer cells [76]. Consequently, a decrease in RORα levels fosters the increased viability of tumor cells which can be counteracted by AMPK. Cancer cells can modulate regulation of RORs as a potential mechanism to evade apoptosis, thereby fostering their proliferation and growth. The mechanism by which cancer cells are causing this modulation is an area that requires further investigation.

## 7. The Role of RORs in Fibrosis

RORs play a crucial regulatory role in the mediation of fibrosis by modulating fibroblast activation and extracellular matrix deposition, positioning them as key targets for comprehension and therapeutic intervention in fibrotic diseases. Fibrosis results from disrupted tissue repair mechanisms, leading to an overabundance of matrix proteins that impairs tissue functionality [91]. Conditions such as pulmonary fibrosis, cardiovascular fibrosis, skin fibrosis, systemic sclerosis, and liver fibrosis illustrate this process [91,92,93,94,95]. The pathways involved in fibrogenesis in relation to these disorders can be different from those involved in inflammatory processes [91]. RORs mediate the antifibrotic effects of vitamin D3 hydroxymetabolites on fibroblasts by regulating both collagen production and fibroblast proliferation [96,97]. Specifically, RORγ and RORα are crucial for the fibrosis-reducing actions of these metabolites [96,97]. In murine fibroblasts, CYP11A1-derived vitamin D3 metabolites effectively inhibit fibroblast proliferation and collagen synthesis [97]. However, this effect is reduced in RORγ-deficient mice, highlighting the crucial role of RORγ in the mediation of these outcomes [97]. Additionally, vitamin D3 derivatives also reduce transforming growth factor β (TGF-β)-1-induced collagen synthesis and fibrosis-related gene expression in RORγ expressing fibroblasts, but these effects are reduced in the absence of RORγ [97]. Analysis in human fibroblasts, where RORα and RORγ had been knocked down, confirmed these findings [96]. The inhibition of human fibroblast proliferation and collagen production by vitamin D3 hydroxymetabolites is also critically dependent on RORα and RORγ, as silencing these receptors with siRNA abolishes the antifibrotic effects [96]. This underscores that RORs are required for the action of vitamin D metabolites on fibroblasts, highlighting their role in regulating fibroblast activity and collagen synthesis, beyond the influence of vitamin D receptors.

Beyond their interaction with vitamin D metabolites, RORs also exhibit a direct capacity to modulate fibrotic processes in various tissues, particularly in liver fibrosis where their activation has been linked to significant reductions in fibrogenic activity [98]. Notably, the activation of RORα mitigates hepatic fibrosis in hepatic stellate cells, a condition marked by the excess buildup of extracellular matrix due to chronic liver injury [98]. This has been demonstrated by a reduction in hepatic collagen deposition and fibrogenic markers, including α-smooth muscle actin and collagen type I alpha 1 chain, in mouse models [98]. In addition, RORα suppresses the phosphorylation of SMAD 2 and SMAD3, highlighting its role in the inhibition of the downstream signaling of TGF-β [98]. Conversely, RORs may exert varying effects on fibrosis depending on the disease context [99]. In mouse models of intestinal fibrosis, often used to study conditions such as Crohn’s disease, RORs have been shown to mediate gut fibrosis [99]. Researchers investigating *salmonella*-induced intestinal fibrosis have discovered that the deletion of RORs protected mice from fibrogenesis in the gut [99]. This protection was reflected by reduced pathology within the affected intestinal tissues, along with suppressed collagen deposition and decreased fibroblast accumulation [99]. Additionally, unlike other studies, this study suggests that RORs do not impact the early stages of inflammation or the body’s infection clearance, but, rather, contribute specifically to the development of fibrosis during the chronic phase of the infection [99]. RORs’ regulation of fibroblast activity and fibrosis has significant therapeutic potential, particularly in relation to cardiac diseases. As fibrosis affects a range of tissues in addition to the liver and skin, further investigation into RORs’ role across various pathological contexts may elucidate its potential as a target for anti-fibrotic therapies.

## 8. Therapeutic Modulation of RORs

As transcription factors, RORs can be modulated therapeutically, a field of research that is rapidly advancing. Research not only focuses on comprehending these receptors, but also on manipulating their activity to achieve targeted physiological outcomes. While natural ligands for RORs exist, the development of selective synthetic agonists presents significant therapeutic potential [100]. Natural ROR agonists, such as cholesterol, and synthetic agonists, including neoruscogenin, nobiletin, and SR1078, influence various organ systems [8,18,77,100,101,102,103,104,105,106,107,108,109,110,111,112,113,114,115]. Neoruscogenin increases the expression of hepatic RORα target genes [103], whereas nobiletin has been studied for its effects on the myocardium, integumentary system, adipose tissue, liver, nervous system, and malignancies [8,18,104,105,106,107,108,109,110]. Activation of RORα by nobiletin enhances autophagy, mitigates mitochondrial dysfunction, and prevents cardiac cell death [8]. In patients suffering from cancer, nobiletin-mediated activation of RORα suppresses cancer cell viability and proliferation [18]. In the nervous system, it attenuates inflammation and cytokine expression in patients suffering from neurodegenerative diseases, including the Alzheimer’s-disease-affected cortex [110]. Inverse agonists targeting RORs, such as T0901317 and SR3335, have demonstrated significant effects on the liver, with SR3335 also influencing adipose tissues and the immune system [113,116,117,118]. Additionally, SR1001 and SR2211 have been explored, each exhibiting distinct biological effects [119,120,121,122,123]. While both modulate the immune system in similar, though not identical, ways, SR1001 also influences the exocrine glands and the pancreas [119,120,121,122,123]. Additionally, JNJ-54271074, a RORγt inverse agonist, blocks Th17 differentiation and IL-17 production from memory T cells, illustrating its therapeutic potential in autoimmune diseases [124]. Moreover, Cpd1 has been discovered to reduce IL-17 production in rat models, contributing to decreased inflammatory responses [125]. Another study has shown that, while Cpd1 and Cpd2 suppress Th17 development and cytokine production, they also induce thymic aberrations [126]. Interestingly, while RORC inhibition holds therapeutic potential for autoimmune diseases by blocking Th17 cell development, recent studies have raised concerns about thymic abnormalities, including preneoplastic risks, with prolonged use [126]. These findings emphasize the need for evaluating long-term safety when considering RORC-targeted therapies, especially in gene therapy applications.

Moreover, hydroxylumisterols, used to suppress the proliferation of human skin cells and melanoma cells, exhibit inverse agonistic effects with respect to RORα and RORγ [127]. This suggests that these receptors are potential targets for the modulation of melanoma progression [127]. Similarly, vitamin D hydroxyderivatives and hydroxytachysterol derivatives, which inhibit skin cell growth and promote antioxidant gene expression, also act as inverse agonists of RORα and RORγ [128,129]. The hydroxymetabolites of the vitamin D3 and tachysterol play a key role in the maintenance of skin integrity by protecting against oxidative and DNA damage as well as inflammation, while inhibiting cell proliferation [130]. Their action on RORs indicates that these inverse agonists could significantly influence cancer progression through modulation of ROR activity [130]. Notably, SHR168442 has been identified as an antagonist with immunomodulatory properties [131], and further research may lead to the discovery and development of additional antagonists. More information on the therapeutic modulation of RORs is summarized in Nematisouldaragh et al. (2024) [100], which offers an extensive analysis of ROR agonists and their diverse impacts, providing valuable insights into their potential applications and ongoing research.

Emerging therapeutic approaches have introduced novel strategies with significant potential. One notable development is the use of RORA-based gene therapy, which has demonstrated efficacy in treating retinal degeneration, particularly in relation to age-related macular degeneration and Stargardt disease [132]. In ATP binding cassette subfamily A member 4 (ABCA4) knockout mice, RORA gene therapy has been found to reduce retinal deposits and restore key inflammatory regulators, leading to improved photoreceptor function [132]. In parallel, small molecule modulators of RORs continue to underscore their therapeutic potential across various diseases. However, challenges persist when it comes to optimizing these compounds in terms of efficacy and specificity while minimizing off-target effects [126]. Additionally, the integration of CRISPR/Cas9 technology [133] offers a novel approach to explore the role of RORs in gene regulation and expression, presenting an exciting avenue for future research that could enhance our understanding of ROR-mediated pathways and their therapeutic implications.

## 9. Discussion

The multifaceted regulatory role of RORs in various cellular processes (Table 1) has attracted substantial research interest in recent years, with a focus on a variety of diseases. RORs have been implicated in the modulation of critical processes under basal physiological conditions, which are fundamental for cellular viability and systemic health. In addition to governing the basal state of the cells, RORs also mediate various signaling cascades during pathological conditions. This review highlights the regulatory role of RORs in different signaling cascades, with an emphasis on cardiovascular diseases, diabetes, and neurological diseases. RORα is the most studied isoform, exhibiting key cardioprotective roles within the cardiovascular system, as well as protective mechanisms that extend beyond the cardiac environment to various organs and systems throughout the body. RORα can regulate different proteins to exert a protective response, such as inhibiting apoptosis and decreasing ROS-producing mitochondria in cardiac myocytes. In addition, the impact of RORα is also demonstrated in its capacity to alleviate cardiac dysfunction and remodeling in both in vitro and in vivo models. Notably, the significance of ROR is also supported by various observations of reduced ROR expression levels in association with disease onset, suggesting ROR regulation as an underlying mechanism of various pathologies. Therefore, further studies are needed to elucidate the intrinsic pathways that ROR isoforms govern to better understand the mechanisms by which RORs exert their protective function. Cell death in the various organ systems, such as the heart and neural system, is irreversible, leading to substantial detrimental effects. Hence, additional research is required to better understand ROR-dependent mechanisms in the regulation of cell viability and therapeutic opportunities of targeting RORs [100] as a means of mitigating the damaging effects of oxidative stress in cardiac myocytes, neurons, and immune cells or other cellular conditions where excessive cell death is known to play a dominating role in disease progression.

## Figures and Tables

**Table 1:** Function of ROR isoforms in different organ systems.

Organ System	ROR Subtype	Function
Cardiovascular system	RORα	- Regulates adaptive quality control processes, such as autophagy and mitophagy, which are essential for cardiac health and mitochondrial function under both normoxic and hypoxic conditions [8,23,24]. - Protects against cardiac hypertrophy and heart failure caused by the renin–angiotensin–aldosterone system (ANG II) activation [10]. - RORs play a key role in the regulation of cardiac inflammation by modulating NF-κB, MAPK, and JAK-STAT pathways, repressing proinflammatory mediators such as IL-6 in response to ANG II stress [47]. - Reduced levels during hypoxia increase cardiac cell death due to autophagy disruption, while upregulation of RORα restores autophagic function, reduces ROS production in mitochondria, and enhances cell survival during hypoxia, while also improving mitochondrial OCR [8]. - Loss of RORα increases IL-6 expression, activates STAT3 and NF-κB, and leads to reduced heart contractility and heart failure in hypertrophy models [10]. - In diabetic hearts, RORα regulates antioxidant defense mechanisms, reducing oxidative stress, apoptosis, and autophagic dysfunction, thereby slowing the progression of diabetic cardiomyopathy [11]. - Reduced levels of RORα in patients suffering from diabetic cardiomyopathy disrupts autophagy and antioxidant gene expression, leading to diastolic dysfunction and cardiac remodeling, while its upregulation mitigates these effects [11]. - RORα inhibition causes defects in mitochondrial morphology and function, leading to elevated apoptosis, mitochondrial aggregation, disorganized cristae, and reduced mitochondrial abundance [11,43,44]. - Regulates MaR1, an anti-inflammatory lipid mediator which contributes to the suppression of inflammation in the heart, liver, and renal systems, particularly in relation to conditions like NASH [56,57,58,60].
Nervous system	RORα	- Provides a neuroprotective function by reducing oxidative stress and regulating redox homeostasis in neurons [12]. - Overexpression of RORα decreases ROS and increases neuronal survival, particularly in cells experiencing oxidative damage [12]. - RORα displays strong antioxidative properties, with its isoform, RORα1, showing neuroprotective effects against redox stresses [36]. - In patients suffering from conditions like ASD, the expression of RORα and its target genes is significantly reduced, indicating its potential involvement in neurodevelopmental regulation [13,14]. - RORα plays a role in the protection of cortical neurons from apoptosis caused by neurotoxic agents such as Aβ, c_2_-ceramide, and H_2_O_2_ by upregulating antioxidant proteins like peroxiredoxin 6 and glutathione peroxidase [36]. - The protective role of RORα extends to neurodegenerative conditions by minimizing ROS production and preventing apoptosis in neurons exposed to oxidative stress [12,13,14,36]. - RORα’s modulation of antioxidant defense mechanisms ensures neuronal resilience under oxidative stress, contributing to its potential role in therapeutic strategies for neurodegenerative diseases [11,36]. - Reduced RORα levels in neurons are associated with increased vulnerability to oxidative damage, leading to impaired neuronal function and cell death [12,36]. - RORα influences neuroinflammation and oxidative stress pathways, suggesting it is a key modulator in neurological disorders involving both neurodegeneration and developmental abnormalities [12,13,14,36].
Immune system	RORα and RORγ	- RORγ is a key regulator of the immune system, playing a critical role in lymphoid organogenesis, thymocyte survival, and the suppression of carcinogenesis and tumorigenesis [3,15,16,17]. - Loss of RORγ decreases the antiapoptotic factor Bcl-xL and the precursor cells in lymphoid organs, leading to the development of lymphoblastic lymphoma [15]. - RORγt, a crucial isoform of *RORC*, is highly expressed in thymocytes, T helper cells, and lymphoid cells, and is required for T cell lineage specification, T helper cell differentiation [49,50,51,52,54], and lymphocyte development [49,50,51,52,54]. - RORγ is necessary for the organogenesis of secondary lymphoid tissues (lymph nodes and Peyer’s patches), regulation of thymopoiesis, immune homeostasis, and adaptive immune responses [7,16,51,52,53,54]. - Downregulation of RORγ expression is observed in aggressive basal-like breast cancer and bladder cancer cells [3,18]. - Activation of RORγ enhances antitumor properties in TNBC, tumor invasion, and growth by suppressing the NF-κB signaling pathway [18]. - RORC is involved in the regulation of cellular division and glucose metabolism [3]. - RORC suppresses cancer cell proliferation and enhances cell apoptosis by inhibiting STAT3 translocation to the nucleus via the PD-L1/ITG β6 signaling cascade [3]. - RORα promotes apoptosis in cancer cells by stabilizing p53 and by selectively binding to other key tumor suppressor genes such as p21, SEMA3F, and FBXW7 [76,88]. - Overexpression of RORα decreases inflammatory cytokine expression and attenuates NASH by increasing the expression of antioxidant enzymes like SOD2 and GPx1 in core immune cells, hepatocytes, and Kupffer cells [39]. - RORα regulates the polarization of macrophages (M1/M2 polarization) [56,64]. - RORα inhibits cell proliferation and migration in cSCC and decreases tumor invasiveness and progression in both human and mouse cSCC cell lines [69]. - ROR expression levels are downregulated in various tumor tissues, including bladder cancer, breast cancer, colorectal cancer, gastric cancer, hepatocellular carcinoma, and cSCC [3,37,69,76,88,89].
Digestive system	RORα	- RORα plays an important role in the maintenance of lysosomal acidity and autophagic function in hepatocytes [37]. - The absence of RORα decreases lysosomal acidity, leading to the aggregation of LC3-II, p62, and NBR1 proteins in hepatocytes, while administration of an adenoviral vector encoding RORα can rescue the lysosomal pH [26]. - RORα upregulates lysosomal genes such as *Atp6v1g1*, which codes for a subunit of vacuolar H^+^-ATPase, essential for maintaining lysosomal activity [26]. - RORα levels are significantly reduced in individuals with liver steatosis, affecting mitochondrial quality control and the oxidative stress response [37,46]. - Induction of RORα enhances mitochondrial quality control and reverses fission processes by upregulating mitochondrial fission proteins [46]. - Knockout of RORα in HFD-fed mice reduces the expression of OXPHOS proteins such as NDUFB8 and ATP synthase, impairing mitochondrial function in the liver [46]. - Overexpression of RORα restores mitochondrial function, increasing fission and oxidative phosphorylation in the liver [46]. - RORα has antioxidative properties in the liver, reducing ROS and lipid peroxidation, and increases the expression of antioxidant defense enzymes such as SOD2 and GPx1 [39]. - Activation of RORα diminishes diethylnitrosamine-induced acute liver injury by regulating mitochondrial dynamics and the antioxidant response [39]. - In liver diseases such as NASH, RORα plays a key role in the reduction of inflammation. MaR1 binds to RORα in liver macrophages, contributing to the suppression of inflammatory processes [56]. - Overexpression of RORα decreases inflammatory cytokine expression and attenuates NASH by upregulating SOD2 and GPx1 in core immune cells, hepatocytes, and Kupffer cells [39]. - RORα alleviates hepatic fibrosis in hepatic stellate cells, leading to reduced hepatic collagen accumulation, and reduces the levels of fibrogenic markers such as α-smooth muscle actin and collagen type I alpha 1 chain [98]. - RORα inhibits the phosphorylation of SMAD 2 and SMAD3 in hepatic stellate cells, underscoring its role of suppressing the downstream signaling of TGF-β [98].
Respiratory system	RORα	- RORα is associated with autophagy regulation in lung diseases, showing a significant role in human lung pathology [28,30,31,32]. - In patients with COPD, increased RORα expression is linked to autophagy-dependent necroptosis and heightened DNA damage in lung tissues, suggesting a role in the pathogenesis and progression of the disease [28,32]. - Dysregulation of autophagy, whether through its increase or decrease, can have detrimental and varying effects across different organs. In the lungs, upregulation of autophagy is particularly harmful and is associated with cellular damage and disease progression [28,30,31,32]. - The absence of RORα inhibits autophagy and autophagosome formation in the lung epithelial cells, highlighting its critical role in maintaining cellular degradation processes [33] - RORα may have a dual role in lung diseases; while it can modulate autophagy to promote cell survival, it may also induce autophagy-dependent necroptosis and modulate autophagy to induce cell death [28,32,33]
Renal system	RORα	- RORα is downregulated in proximal tubules, the cortex, and the outer medulla of the kidney during renal I/R injury, post I/R and in diabetes-related renal damage [34] - The absence of RORα leads to increased oxidative stress, elevated epithelial cell apoptosis, and exacerbated renal tubular injury following I/R injury [34,35] - RORα deficiency worsens renal I/R injury, leading to endoplasmic reticulum stress, impaired mitochondrial function, and reduced autophagic activity, which contribute to greater renal dysfunction and increased kidney morphological damage [34] - Upregulation of RORα, achieved through pharmacological agonists, enhances renal cell viability, reduces epithelial cell apoptosis, and suppresses oxidative stress in ischemic kidneys [34] - Protective role in diseases related to the gastrointestinal tract, including bowel diseases and ulcerative colitis [5] - In bowel diseases, RORα reduces inflammation by interacting with NF-κB and the corepressor HDAC3, though this mechanism may influence broader systemic inflammatory responses [5,85]
Integumentary system	RORα and RORγ	- RORα is involved in the inflammatory process and neutrophil recruitment in the integumentary system [61] - RORα’s induction of 12-LOX leads to elevated levels of MaR1, which plays a key role in resolving skin inflammation and promoting tissue repair [56] - RORα activation helps repress skin edema and decreases inflammation in skin injury models subjected to UVB exposure through upregulation of MaR1 [56,61] - By regulating MaR1 levels, RORα inhibits cytokine production and neutrophil recruitment, thus modulating the immune response and reducing excessive inflammation in the skin [56,61]. - RORα and RORγ modulate the anti-fibrotic effects of vitamin D3 hydroxymetabolites on fibroblasts by regulating both collagen production and fibroblast proliferation [96,97].
